# Case Report: RYR1-related myopathy with hypoxic ischemic encephalopathy—a case of severe neonatal presentation due to a *de novo* variant of uncertain significance

**DOI:** 10.3389/fped.2026.1875252

**Published:** 2026-09-03

**Authors:** Saptadweepa Sanghamitra, Rupashree Mandala, Hetal Budh, Yashita Arora, Pedro Rivera-Hernandez, N Ja Hpa, Valerie Elberson, Munmun Rawat, Srinivasan Mani

**Affiliations:** Division of Neonatology, Department of Pediatrics, University at Buffalo, Buffalo, NY, United States

**Keywords:** hypoxic ischemic encephalopathy, infant, newborn, RYR1-related myopathy, variant of uncertain significance

## Abstract

Pathogenic variants in the Ryanodine Receptor 1 (RYR1) gene represent the most common cause of congenital myopathy. The severity of RYR1-related myopathy presenting in the neonatal period is quite variable, ranging from a perinatal lethal type to a benign late-onset form with progressive improvement. We report a newborn infant who presented at birth with severe hypoxic ischemic encephalopathy (HIE), underwent therapeutic hypothermia (TH), and was later diagnosed with RYR1-related myopathy. Exome sequencing identified a *de novo* variant of uncertain significance (VUS) in the RYR1 gene (c.14564T>G). The clinical course of the patient was complicated by severe generalized hypotonia, persistent respiratory failure requiring mechanical ventilation, hypocalcemia, chylothorax, and eventual redirection of care with death at 1 month of life. The infant's phenotype included congenital fractures, arthrogryposis, macrocephaly, and cryptorchidism. The phenotype overlapped with the clinical findings of RYR1-related myopathy, providing evidence supportive of the pathogenicity of the identified VUS. Our report aims to be the first step in generating evidence for the reclassification of this VUS. We also review the literature on the spectrum of neonatal presentations and outcomes of RYR1-related myopathy, with particular emphasis on HIE, and discuss the role of TH in infants with underlying congenital or genetic disorders.

## Introduction

The Ryanodine Receptor 1 (RYR1) gene, located on chromosome 19, encodes the ryanodine receptor present in the sarcoplasmic reticulum of skeletal muscle (1). The ryanodine receptor functions as a calcium release channel and plays a central role in excitation–contraction coupling. Pathogenic variants in this gene are the most common cause of congenital myopathy, previously classified as central core myopathy, multiminicore myopathy, central myofiber-type disproportion, centronuclear myopathy, and nemaline myopathy based on muscle biopsy findings. The severity of RYR1-related myopathy (RYR1-RM) presenting in the neonatal period is quite variable, ranging from a perinatal lethal type to a benign late-onset form with progressive improvement (2). In infants with severe presentation at birth, respiratory failure due to RYR1-RM at birth causes severe hypoxia and could lead to hypoxic ischemic encephalopathy (HIE).

We report a patient with severe RYR1-RM who presented at birth with severe HIE, respiratory failure, congenital fractures, arthrogryposis, macrocephaly, cryptorchidism, and later developed chylothorax. Our patient had a heterozygous *de novo* missense variant of uncertain significance (VUS) p. (Val4855Gly) (GTG>GGG): c.14564T>G, located in exon 101 of the C-terminal region of the RYR1 gene (NM_000540.3) (3). This is a novel variant not observed at significant frequency in large population cohorts and has not been previously reported as pathogenic or benign. The framework for variant classification of Mendelian disorders into pathogenic, likely pathogenic, uncertain significance, likely benign, and benign is based on the strength of evidence from a combination of specific criteria outlined in the revised American College of Medical Genetics and Genomics/Association of Molecular Pathology (ACMG/AMP) guidelines (4). These criteria were established using typical variant evidence from data sources such as population frequencies, in silico predictions, functional studies, segregation analyses, and phenotypic data. The guidelines emphasize the importance of reanalysis and reclassification based on evolving evidence on variants.

Our report aims to be the first step in generating evidence for the reclassification of this VUS. In addition, we review the literature on the spectrum of neonatal presentation and outcomes of RYR1-RM. Moreover, evidence for therapeutic hypothermia (TH) in infants with HIE and an underlying congenital or genetic disorder is limited. We also aim to explore this knowledge gap by highlighting the potential pitfalls in the diagnosis of HIE and weighing the risks and benefits associated with TH in infants with underlying congenital or genetic disorders.

## Case presentation

### Prenatal and birth history

Our patient was a late preterm male infant, born at 35 week and 3 days of gestation to a 26-year-old gravida 2 para 1 mother, whose pregnancy was complicated by polyhydramnios and anemia. The mother's blood group was A positive, and her routine prenatal screening yielded normal results. The infant's mother was referred to our hospital's labor and delivery department with a biophysical profile of 4/10. A repeat cesarean section was performed urgently under spinal anesthesia. Amniotic fluid was clear. The infant was born with a birth weight of 2.390 kg (34.5th percentile), length of 47 cm (60th percentile), and head circumference of 36.5 cm (99.8th percentile). At birth, the infant was apneic, floppy, and cyanotic. Deferred cord clamping was not performed. Umbilical arterial blood gas revealed a pH of 7.2 and a base excess of −1.4. The infant needed endotracheal intubation and positive pressure ventilation in the delivery room for stabilization. Apgar scores were 1 at 1 min, 3 at 5 min, and 4 at 10 min.

### Initial management

The infant was admitted to our Level IV neonatal intensive care unit (NICU). On admission, physical examination revealed macrocephaly, widely spaced eyes, prominent forehead, frontal bossing, micropenis, upper-extremity joint contractures, and bilateral hypotonia. The infant was noted to have a swollen left thigh, suspicious for fracture of the left femur, which was subsequently confirmed radiographically. The infant had diffuse petechiae over the chest and extremities. Neurological examination revealed an unresponsive infant with no spontaneous movements, hypotonia, and absent reflexes. Venous blood gas obtained within an hour of birth showed mixed respiratory and metabolic acidosis (pH 7.11, pCO_2_ 65 mm Hg, base excess of – 9). The infant was classified as having severe HIE based on Sarnat staging. A non-contrast computed tomography (CT) of the brain was performed soon after admission. It revealed enlargement of extra-axial cerebrospinal fluid spaces, which was suspected to represent an early presentation of benign enlarged extra-axial spaces of infancy. There was no evidence of hydrocephalus. Fluid was noted within the mastoid air cells bilaterally.

**Figure 1:**
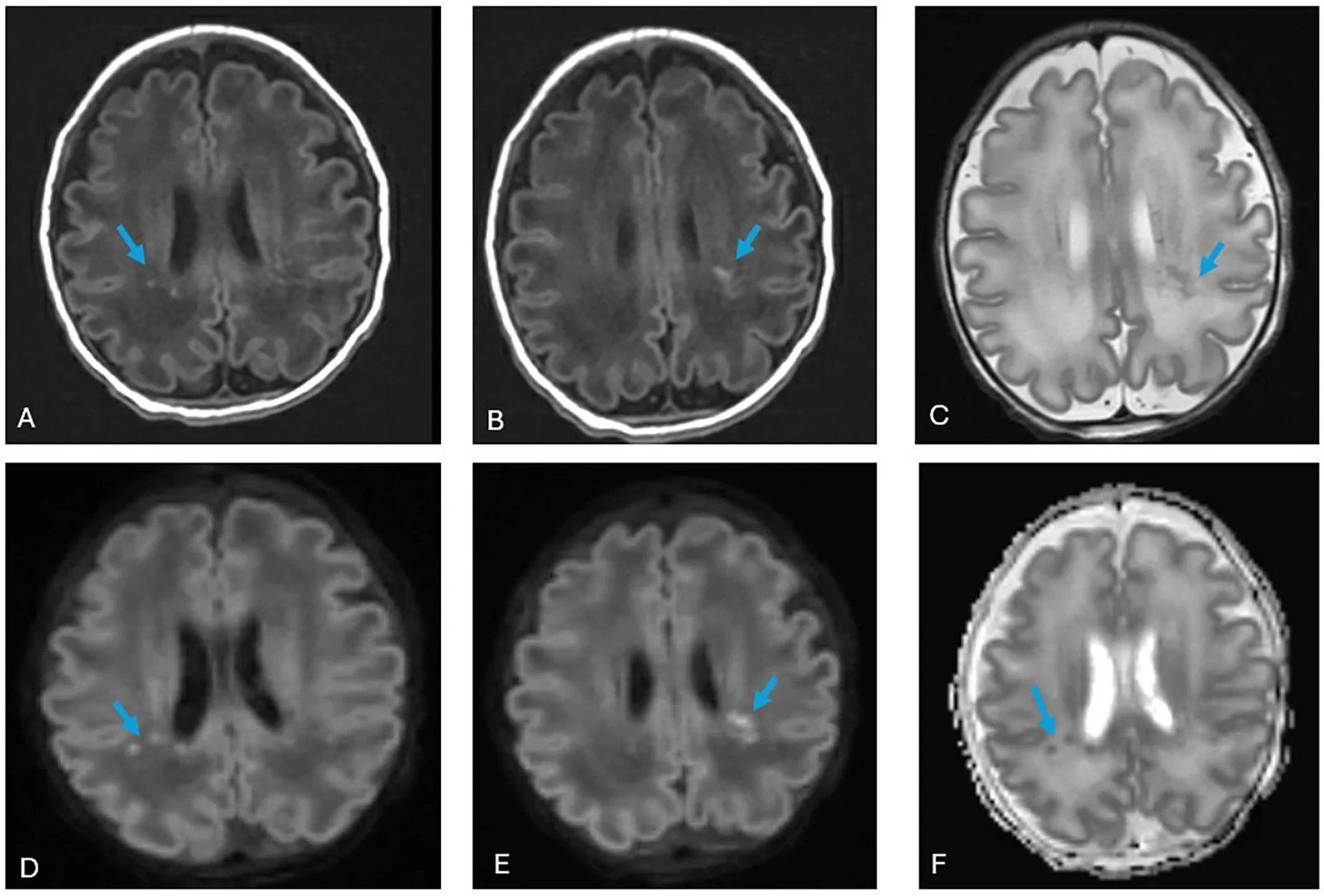
Non-contrast MRI of the brain shows multifocal punctate restricted diffusion (blue arrows) in the posterior white matter of the bilateral cerebral hemispheres, suggestive of scattered bilateral white matter hypoxic ischemic injury. Panels **(A,B)** are axial T1-weighted images, Panel **(C)** is an axial T2-weighted image, Panels **(D,E)** are diffusion-weighted images (DWI), and Panel **(F)** is a diffusion ADC (apparent diffusion coefficient) image. Note that the areas of restricted diffusion are hyperintense in DWI and hypointense in the ADC map.

TH was initiated for severe HIE based on Sarnat staging. The infant's initial chest X-ray was suggestive of respiratory distress syndrome due to surfactant deficiency of prematurity. Considering the high oxygen requirement in a late preterm infant with risk of surfactant inactivation associated with perinatal asphyxia, exogenous surfactant was administered. The infant remained on mechanical ventilation. Intravenous antibiotics were started for suspected sepsis but discontinued after 24 h because of negative blood culture. Initial laboratory tests showed a deranged coagulation profile, necessitating transfusion of fresh-frozen plasma, which resulted in improvement. The infant was monitored on continuous video electroencephalogram (EEG) for seizures during the TH period (72 h). EEG demonstrated excessive discontinuity, diffuse dysrhythmia, and an ill-differentiated wake/sleep pattern, consistent with underlying encephalopathy. No clinical or EEG seizures were observed. The infant was rewarmed on day of life (DOL) 3. He underwent magnetic resonance imaging (MRI) of the brain, which showed multifocal punctate restricted diffusion in the posterior white matter of the bilateral cerebral hemispheres, consistent with scattered bilateral white matter hypoxic ischemic injury (Figure 1). Globally prominent extra-axial cerebrospinal fluid spaces were seen, as noted in the prior CT brain scan.

### Course during hospitalization

The infant remained on mechanical ventilation throughout the hospitalization, alternating between conventional ventilation and high-frequency oscillatory ventilation (HFOV). Multiple attempts at extubation failed, with apnea documented as the primary cause of failure. On DOL 11, the infant's respiratory status worsened and he was diagnosed with aspiration pneumonia on evaluation. His condition improved with intravenous antibiotics and supportive care. Echocardiography was conducted, revealing no significant abnormalities.

Severe hypocalcemia was noted soon after birth, requiring treatment. Pediatric endocrinology was consulted, and the infant was evaluated for hypoparathyroidism, vitamin D deficiency, and hypercalciuria. Tests revealed low 25-hydroxyvitamin D (19 ng/mL), high 1,25-dihydroxyvitamin D (207.3 pg/mL), and hyperphosphatemia (9.7 mg/dL). Serum parathormone levels were inappropriately normal (61 pg/mL) in the clinical setting of hypocalcemia due to an immature parathyroid gland response in the preterm infant. Prematurity-related transient hypoparathyroidism, vitamin D insufficiency, and perinatal asphyxia were considered contributory to hypocalcemia. The infant was managed with cholecalciferol, calcium, and magnesium supplements, initially via total parenteral nutrition and then enterally once the infant reached full feeds on DOL9. Subsequently, the serum calcium levels showed gradual improvement. The infant continued to develop pathological fractures due to osteopenia, despite gentle handling and adherence to fracture-preventive strategies. A scrotal ultrasound was performed, which showed an intra-abdominal left testis and a mobile right testis. Plans were made to evaluate the underlying endocrine cause of the micropenis with hormonal testing (testosterone, FSH, and LH) during the mini-puberty phase (between 1 and 3 months of age) to diagnose conditions such as hypopituitarism and congenital hypogonadotropic hypogonadism.

A pediatric genetics consultation was obtained shortly after birth. Once the infant was stabilized, trio exome sequencing (infant and biological parents) was performed on DOL 7, with informed consent from the parents. The genetics and NICU teams met with the family to review the exome sequencing results on DOL18. The parents were informed that a *de novo* variant of uncertain significance had been identified in the RYR1 gene (c.14564T>G), which can be associated with a spectrum of neuromuscular disorders that typically present with severe hypotonia and respiratory insufficiency. The infant's phenotype overlapped with the clinical findings of RYR1-related myopathy. Our patient met the following ACMG/AMP criteria for classifying variants: (1) *de novo* (both maternity and paternity confirmed) in a patient with disease and no family history (PS2); (2) located in a mutational hotspot (PM1); and (3) patient's phenotype or family history is highly specific for a disease with a single genetic etiology (PP4), providing evidence supporting the pathogenicity of the identified VUS. No additional pathogenic variants were identified by exome sequencing that could account for the skeletal, craniofacial, and metabolic changes seen in our patient. The parents were counseled that the variant found in their child was new and not present in either parent, leading to a low likelihood of recurrence in future pregnancies. However, there was a low (<1%) possibility that either parent may have germline mosaicism for this variant, which was not tested. The parents were informed that no specific treatments or cures for RYR1-RM were available and that management would remain supportive.

The pediatric pulmonology service was consulted in the context of chronic respiratory failure due to congenital myopathy for long-term management during the fourth week of hospitalization. It was discussed that the infant would require a tracheostomy and long-term home ventilatory support due to severe hypotonia. Long-term improvement in the respiratory status could not be predicted. A muscle biopsy was planned to better characterize the type of myopathy histologically once clinical stability was achieved. However, given the expected developmental composition of the muscles and the low utility of muscle biopsy or electromyogram results in altering management, further investigations were deferred. The infant was tested to have normal creatine kinase levels (116 unit/L).

**Figure 2:**
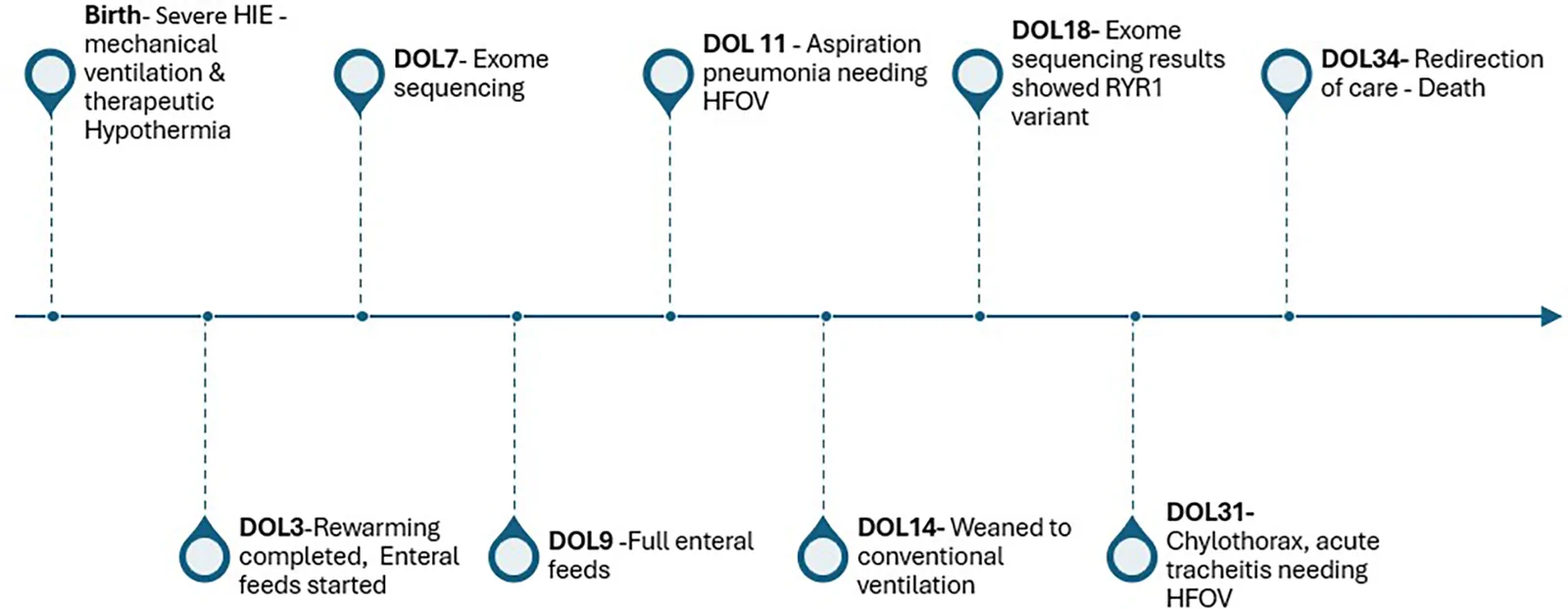
Timeline of events during the course of the infant's hospitalization.

On DOL31, the infant was noted to have developed new-onset right-sided pleural effusion, which was confirmed as chylothorax by elevated pleural fluid triglyceride levels. The infant was managed with pigtail catheter drainage and escalation of support to HFOV. On further sepsis evaluation, the infant was diagnosed with acute tracheitis due to *Stenotrophomonas* and *Serratia*, which were treated with broad-spectrum antibiotics. The infant's clinical status continued to worsen, and the family met with the multidisciplinary team to discuss the prognosis, the dynamics of the infant requiring home ventilation, and the option of redirecting care. The parents decided to redirect their infant's care toward palliation. The infant died following extubation on DOL34. The timeline of the infant's hospital course is summarized in Figure 2.

## Discussion

The RYR1 gene is located on chromosome 19q13.2, spanning more than 159 kb and containing 106 exons (5, 6). It encodes the ryanodine receptor protein present in the sarcoplasmic reticulum and transverse tubules. Ryanodine receptors are the largest known homotetrameric ion channel with a molecular mass of 2.2 MDa (7). There are 5,038 amino acids in each of the monomers with a molecular mass of 568 kDa (1). There are more than 700 variants in the RYR1 gene that have so far been identified (8). The RYR1 variants can be inherited both as autosomal dominant and recessive. There are three mutational hotspot regions (N-terminal, central, and C-terminal) noted in dominantly inherited variants (1). The recessively inherited variants do not exhibit specific hotspot regions. There is a correlation between the variant's mode of inheritance and the histopathological subtype of congenital myopathy. Central core myopathy occurs due to dominantly inherited variants, and multiminicore disease, centronuclear myopathy, and congenital fiber-type disproportion are associated with recessively inherited variants. The literature suggests that the position of the variant in one of the three mutational hotspot regions of the RYR1 gene has phenotype correlates. The variants in the N-terminal and central regions lead to malignant hyperthermia susceptibility, and the variants in the C-terminal region lead to central core disease. Thus, the genotype–phenotype correlation in RYR1-RM is complex, influenced by the mode of inheritance, variant type, and location (9). The variant identified in our patient was in the exon 101 of the C-terminal region.

RYR1 is a cation-gated calcium channel located in the sarcoplasmic reticulum and the transverse tubule in skeletal muscle. The dihydropyridine receptors (DHPR) or CaV1.1 are voltage-gated L-type calcium channels located in the transverse tubule membranes. RYR1 and DHPR are coupled to each other physically (7). DHPR senses the depolarization and undergoes conformational change that triggers the opening of the RYR1 channel and calcium release, leading to muscle contraction. The RYR variants disrupt the function of the channel by causing abnormal calcium release, leading to an imbalance in the intracellular calcium homeostasis, which results in excitation–contraction uncoupling and muscle weakness (1). In our patient, the protein variation effect analyzer (Provean) was used to assess the impact of amino acid substitution (valine replaced by glycine). This in silico tool yielded a delta alignment score of −6.24, indicating that the amino acid change due to the missense mutation had a deleterious effect on the protein structure and function.

RYR1-RM is the most common cause of congenital myopathy, with a point prevalence of 1:90,000 in the pediatric age group in the US (1). Neonatal RYR1-RM presents across a wide spectrum. On the most severe end, patients can present with lethal multiple pterygium syndrome or fetal akinesia syndrome. On the milder end of the spectrum, patients may follow a benign course with progressive improvement, leading to independent ambulation, absence of need for respiratory support, and normal speech and swallowing (10). In a European retrospective cross-sectional cohort of congenital myopathies, 44.4% (44/99) of patients were diagnosed with RYR1-RM, with 100% survival without the need for long-term ventilatory support (11). This cohort included neonates presenting with severe disease onset. They found a good genotype–phenotype correlation across the different genetic causes of congenital myopathies. Infants with recessively inherited RYR1-RM had more frequent neonatal onset and severe disease at birth with bulbar and respiratory complications. The risk of gastrostomy/jejunostomy tube placement was higher in recessively inherited RYR-RM (35%) compared with none in the dominantly inherited disease.

Our review of the case reports and the case series reporting neonatal RYR1-RM showed that generalized severe hypotonia and respiratory failure needing persistent ventilatory support are the most common presenting findings, similar to our patient. Ptosis, ophthalmoplegia/ paresis, bulbar weakness manifesting as suck and swallow dysfunction needing gastrostomy tube placement, and scoliosis are the other commonly reported findings. Infants with congenital myopathies have low bone mineral density, predisposing them to pathological long-bone fractures (12). However, we considered the biochemical derangements observed in our patient's evaluation of hypocalcemia as unrelated to RYR1-RM. The rarer presentations associated with RYR1-RM reported in each study, along with age at presentation, age at diagnosis, severity, and outcomes, are presented in Table 1 (13–25). The phenotypic features noted in our patient, such as macrocephaly, hypertelorism, and frontal bossing, are not characteristic of RYR1-RM but have been reported previously (26). Common muscle biopsy findings suggestive of RYR1-RM include the presence of cores, fiber-type uniformity or type 1 predominance, and increased internal or central nuclei (27). Muscle biopsy findings may not be reliably detected in the neonatal period and may evolve over time. The MRI of the quadriceps femoris in the thigh may show T2 hyperintense signals with characteristic sparing of the rectus femoris. Electromyography may reveal a myopathic pattern. Our patient did not undergo muscle biopsy or MRI of the thigh muscles to confirm the diagnosis. The absence of histological and imaging findings to corroborate the genetic and phenotypic findings is a limitation of our report.

**Table 1:** Literature review of RYR1-related myopathy with neonatal presentation.

Study	Type	Gestational age at birth (weeks)	Age at diagnosis	Rare clinical presentation reported	Significance of the report	Age at reporting	Severity of the disease	Outcome
Monnier et al. (13)	Case report	NR	2 months	None	First report of genomic rearrangement in the RYR1 gene	2 months	Severe	Death
Bevilacqua et al. (14)	Case series	NR	4 months	None	Muscle biopsy findings mimic centronuclear myopathy	NR	Moderate	Progressive improvement in motor function
Bharucha-Goebel et al. (15)	Case series (*n* = 11)^a^	NR	NR	Cleft palate, congenital rigid spine	Sparing of the rectus femoris in muscle MRI	NR	Variable	Independent ablution, no intellectual impairment
Laforgia et al. (16)	Case report	34	12 days	Dysmorphic facies—low-set ears with dysplastic lobes, dysmorphic antihelix, anteverted nostrils, arachnodactyly, low-set thumbs, bilateral clinodactyly; fracture of the femur and humerus	Hypomorphic variant with a missense variant causing RYR1-RM	1 month	Severe	Death
Hayakawa et al. (17)	Case report	36 + 3/7	NR	Transient ectopic atrial tachycardia	Association of sinus node dysfunction	11 months	Severe	Need for tracheostomy, home respirator, gastrostomy tube
Tanaka et al. (18)	Case report	NR	15 days	Chylothorax	Association of centronuclear myopathy and congenital chylothorax	4 years	Severe	Ventilator dependent
Mauri et al. (19)	Case series (*n* = 4)^a^	34–37	5 days to 5 years	None	Role of muscle biopsy and MRI in aiding genetic testing	3 years to 8 years	Severe	Independent ambulation to the need for tracheostomy with home ventilation and gastrostomy feeding
Biancalana et al. (20)	Case series (*n* = 6)^a^	NR	1–4 years	Fetal macrosomia, high arched palate, pes planus, hallux valgus	Severe initial presentation with a benign course with progressive improvement	3–20 years	Benign	Progressive improvement in the first decade
Shillington et al. (21)	Case report	38	28 days	Dysmorphic facies, transposition of great vessels	Association with congenital heart disease, brain, and retinal hemorrhages	1 month	Severe	Redirection of care
Baker et al. (22)	Case report	NR	71 days	Pulmonary hypertension, eventration of the diaphragm	Early rapid exome or genome sequencing as a first-line test	5 months	Severe	Death
Etarhuni et al. (23)	Case series (*n* = 1)^a^	NR	NR	Cleft palate, undescended testis	The onset and severity of disease presentation varied between families	3 years	Severe	Profound motor delay and gastrostomy feeding
Janßen et al. (24)	Case report	NR	6 years	None	Severe disease present in patients with intronic splice variants	6 years	Severe	Ventilator dependent
Patial et al. (25)	Case report	38	1 month	Hypoxic ischemic encephalopathy (HIE)	HIE masquerades as congenital myopathy	2 months	Severe	Death

NR, not reported.

a Cases with neonatal presentation only were selected from the case series.

HIE is a type of neonatal encephalopathy occurring due to perinatal events that deprive the newborn infant's brain of oxygen or blood flow. Neuromuscular disorders that precipitate respiratory failure at birth may impair the transition from fetal to neonatal circulation, resulting in HIE (28). Congenital myopathies can lead to severe diaphragmatic weakness at birth, leading to respiratory failure. Respiratory failure at birth could predispose the infant to HIE (29). Congenital myopathies do present with severe hypotonia at birth, with absent or weak reflexes and reduced spontaneous activity. This could lead to erroneous classification of an infant as moderate or severe HIE, according to the Sarnat staging criteria (three out of six criteria) used in the evaluation of an infant with HIE for TH. However, the key difference lies in the level of consciousness being affected in myopathic infants who develop HIE secondary to severe respiratory failure at birth. In the absence of HIE, infants with pure neuromuscular disorders remain alert on neurological exam. The neurological immaturity of late preterm infants at birth and its independent influence on the Sarnat staging in HIE were examined in a retrospective study (30). The findings indicated that late preterm infants, compared with term infants, exhibited higher median scores for the Moro reflex and autonomic respiratory score; however, overall examination scores, including level of consciousness, did not differ statistically. The study concluded that the likelihood of overdiagnosing HIE in late preterm infants based solely on their neurological immaturity is minimal. Our patient met the criteria for TH—abnormal biophysical profile leading to delivery, need for respiratory support for >10 min at birth, and pH in the range of 7.01–7.15 or base deficit in the range of 10–15.9 mmol/L on a blood sample obtained within 60 min of birth. Since postnatal respiratory failure and the associated severe hypoxia at delivery were the causes of HIE, the cord blood gas in our patient was near normal, and acidosis was noted only in the first postnatal blood gas. Notably, our patient's level of consciousness was classified as “unresponsive” during the neurological examination, the distinguishing feature pointing to HIE in a myopathic infant as discussed earlier. Neuroimaging and EEG of our patient correlated with perinatal hypoxic ischemic injury. A retrospective study that investigated the brain injury pattern in late preterm infants with HIE found that white matter/cortex injury is the most frequent form of injury, affecting nearly half of the study cohort (31). Diffusion restriction in the thalamus, cerebellum, optic radiation, and corpus callosum was also commonly observed. The punctate white matter lesions were significantly more common in late preterm infants compared to term infants with HIE treated with TH (17.6% vs. 1.3%). However, clinical context is important while interpreting punctate white matter abnormalities because they are generally more common in moderate-to-late preterm infants (32). We considered the prominent extra-axial cerebrospinal fluid spaces noted in our patient as a benign incidental finding unrelated to HIE or other structural abnormalities (33). Seizures are a common complication of HIE, with reported incidence as high as 50%. Studies have shown that TH is associated with a reduced seizure burden in infants with moderate HIE (34). EEG seizures were unusually absent in our patient. There are similar reports of infants with HIE and underlying congenital myopathy in the literature (25, 35)—one due to intranuclear rod myopathy and the other due to RYR1 myopathy. Interestingly, both cases were marked by a diagnosis of HIE without a known perinatal sentinel event.

Infants with congenital myopathy have characteristic sparing of brain function, and the survivors often achieve normal brain development in the absence of HIE. In such a scenario, TH, the only proven treatment available for HIE, could be offered to infants with suspected myopathy. This is especially useful in the subgroup of patients in whom the RYR1-RM—despite its severe neonatal presentation—could gradually improve to normal cognitive function. In patients with persistent severe myopathic phenotypes, TH may not alter the long-term outcome, as was the case for our patient. TH is not without risks. The common adverse effects associated with TH are sinus bradycardia, prolongation of the QT interval on the electrocardiogram, systemic hypotension requiring inotropes, and persistent pulmonary hypertension (28). A small case series of TH in late preterm infants with HIE reported hypofibrinogenemia as a significant risk (36). Given the potential harm related to TH, the risks and benefits of this treatment should be discussed with the family on a case-by-case basis for infants born at 35 0/7–35 6/7 weeks’ gestation (37). There is a lack of good-quality evidence to inform clinicians regarding the risks and benefits of TH for HIE in neonates with congenital birth defects or neuromuscular disorders (38). Developing evidence based on meta-analyses of smaller observational studies and expert consensus could help inform practice. Such efforts would be especially helpful in the management of HIE in infants with underlying congenital or genetic disorders, such as tracheo-esophageal fistula, gastroschisis, congenital diaphragmatic hernia, and spinal muscular atrophy (39).

### Parental perspectives

At the time of NICU admission, the parents expressed a strong desire for full resuscitation and life-saving interventions. Their decision was rooted in deeply held cultural and religious values. The parents were shocked and unprepared to come to terms with the severity of their baby's condition at birth. The parents expressed significant emotional distress upon learning about the multiple long-bone fractures and the possibility of underlying congenital or genetic etiologies. The infant had episodic blinking of eyes and occasional involuntary muscle contractions that were interpreted by the parents as signs of neurological recovery, creating a false sense of hope to continue intensive medical care. The parents expressed a desire to be present at the bedside during extubation attempts and were disappointed by the failure of those attempts. Despite the use of a professional interpreter, there was concern that the parents did not fully understand the patient's condition due to a language barrier.

The prolonged intensive care without meaningful recovery and the continued need for escalation of supportive treatment were observed by the parents at the bedside, and they slowly began to reflect on their management choices. The results of exome sequencing gave them a sense of clarity to understand the nature of their child's disease. The parents understood that the diagnosed condition did not have a cure, which slowly modified their expectations of treatment. The parents expressed a desire to avoid chest compressions, considering the risk of rib fractures. The parents’ cultural and religious beliefs were taken into consideration during communication. One of our team members, who spoke the parents’ native language, was invited to participate in the family meetings, in addition to the professional language interpreter. We gave the family a medical summary that they shared with a family physician in their home country, which helped build trust in the information we provided. The parents stated that their discussion with a religious community leader in their home country over the phone helped them decide to redirect care.

## Conclusion

Our patient with a novel *de novo* VUS in the RYR1 gene presented with a severe form of RYR1-RM complicated at birth by HIE. The VUS demonstrated good phenotypic correlation, and *in silico* analysis indicated that the missense mutation had a deleterious effect on the protein structure and function, providing evidence supportive of pathogenicity. However, the absence of histological confirmation of congenital myopathy limits the certainty of our diagnosis. Considering the severe presentation of RYR1-RM, the absence of proven treatment options, and progressive worsening on supportive care, the parents chose to redirect care, and the infant died at 1 month of life. This report highlights the need for future research to determine the risks and benefits of TH for HIE in infants with suspected or known congenital/genetic disorders at birth.

## Data Availability

The raw data supporting the conclusions of this article will be made available by the authors, without undue reservation.
